# DNA-dependent protein kinase catalytic subunit modulates the stability of c-Myc oncoprotein

**DOI:** 10.1186/1476-4598-7-32

**Published:** 2008-04-22

**Authors:** Jing An, Dong-Yan Yang, Qin-Zhi Xu, Shi-Meng Zhang, Yan-Ying Huo, Zeng-Fu Shang, Yu Wang, De-Chang Wu, Ping-Kun Zhou

**Affiliations:** 1 Department of Radiation Toxicology and Oncology, Beijing Institute of Radiation Medicine, Beijing, 100850, P R China

## Abstract

**Background:**

C-Myc is a short-lived oncoprotein that is destroyed by ubiquitin-mediated proteolysis. Dysregulated accumulation of c-Myc commonly occurs in human cancers. Some of those cases with the dysregulated c-Myc protein accumulation are attributed to gene amplification or increased mRNA expression. However, the abnormal accumulation of c-Myc protein is also a common finding in human cancers with normal copy number and transcription level of c-Myc gene. It seems that the mechanistic dysregulation in the control of c-Myc protein stabilization is another important hallmark associated with c-Myc accumulation in cancer cells. Here we report a novel mechanistic pathway through which DNA-dependent protein kinase catalytic subunit (DNA-PKcs) modulates the stability of c-Myc protein.

**Results:**

Firstly, siRNA-mediated silencing of DNA-PKcs strikingly downregulated c-Myc protein levels in HeLa and HepG2 cells, and simultaneously decreased cell proliferation. The c-Myc protein level in DNA-PKcs deficient human glioma M059J cells was also found much lower than that in DNA-PKcs efficient M059K cells. ATM deficiency does not affect c-Myc expression level. Silencing of DNA-PKcs in HeLa cells resulted in a decreased stability of c-Myc protein, which was associated the increasing of c-Myc phosphorylation on Thr58/Ser62 and ubiquitination level. Phosphorylation of Akt on Ser473, a substrate of DNA-PKcs was found decreased in DNA-PKcs deficient cells. As the consequence, the phosphorylation of GSK3 β on Ser9, a negatively regulated target of Akt, was also decreased, and which led to activation of GSK 3β and in turn phosphorylation of c-Myc on Thr58. Moreover, inhibition of GSK3 activity by LiCl or specific siRNA molecules rescued the downregulation of c-Myc mediated by silencing DNA-PKcs. Consistent with this depressed DNA-PKcs cell model, overexpressing DNA-PKcs in normal human liver L02 cells, by sub-chronically exposing to very low dose of carcinogen 2,3,7,8-tetrachlorodibenzo-p-dioxin (TCDD), increased c-Myc protein level, the phosphorylation of Akt and GSK3 β, as well as cell proliferation. siRNA-mediated silencing of DNA-PKcs in this cell model reversed above alterations to the original levels of L02 cells.

**Conclusion:**

A suitable DNA-PKcs level in cells is necessary for maintaining genomic stability, while abnormal overexpression of DNA-PKcs may contribute to cell proliferation and even oncogenic transformation by stabilizing the c-Myc oncoprotein via at least the Akt/GSK3 pathway. Our results suggest DNA-PKcs a novel biological role beyond its DNA repair function.

## Background

The c-Myc oncoprotein is a short-lived basic helix-loop-helix leucine-zipper transcription factor that, together with its dimerization partner Max, binds to specific E-box sequences and is responsible for controlling a set of genes whose functions impinge directly upon the machinery of cell growth and proliferation [1,2]. C-myc has the transforming capacity, even the activation of the c-Myc gene alone can lead to the formation of liver cancers and inactivation of the c-Myc is sufficient to induce sustained regression of invasive liver cancers [3]. Dysregulated accumulation of c-Myc oncoprotein commonly occurs in various human cancers (30–50%) [4-9], and in most cases is associated with disease progression.

Proteolysis of c-Myc protein within minutes of its synthesis occurs through the ubiquitin-proteasome pathway [10], which involves the F box protein and the ubiquitin ligase components, Skp2 and Fbw7 [11-15]. The c-Myc transactivation domain (TAD), spanning amino acids 40–150, contains the sequence PTPPLSP (residues 57–63), within which both T58 and S62 are phosphorylated. The critical phosphorylation event of T58 and S62 determines the protein half life [16]. The phosphorylation of S62 mediated by the Ras/MEK/ERK kinase pathway, is believed to be a prerequisite for the phosphorylation of T58 regulated through the phosphatidylinositol 3-kinase/Akt (PKB)/glycogen synthase kinase 3 (GSK3) pro-survival pathway [7,17,18]. Phosphorylation of c-Myc on T58 by GSK3 regulates the binding of Fbw7, which in turn triggers c-Myc ubiquitination and degradation [15].

Mechanisms for the dysregulated accumulation of c-Myc protein in cancers, as well as the means by which c-Myc stimulates cell proliferation and transformation, have received much attention. Indeed, a number of studies demonstrated that T58 mutation occurred in some cancers, which resulted in decreased ubiquitination and proteolysis of c-Myc [17-19]. However, the abnormal accumulation of c-Myc protein is also a common finding in human cancers with intact and normal copy or expression levels of the c-Myc gene, suggesting the mechanistic dysregulation in the control of c-Myc protein stabilization in human cancers.

DNA-dependent protein kinase catalytic subunit (DNA-PKcs) is a member of a sub-family of proteins containing a phosphoinositol (PI) 3-kinase domain with the activity of a serine/threonine protein kinase [20,21]. It is well known that DNA-PKcs is required for the non-homologous end joining (NHEJ) pathway of DNA double-strand breaks, V (D) J recombination of immunoglobulin genes and T cell receptor genes [20], and telomere length maintenance [22,23]. However, overexpression of DNA-PKcs has recently been unveiled in various human cancers [24-30], and its expression level was also reported to correlate with the development of productive tissues or the differentiation and proliferation status of some cell types [31-34]. It is still unclear what the biological significance is for this overexpressed DNA-PKcs in human cancers. Recently we have reported that silencing of DNA-PKcs mediated by specific siRNA molecules led to strongly decreased c-Myc protein level without changing c-myc mRNA expression [35], and increased expression of some of the c-Myc repressing genes, e.g. p21, p27 and NDRG1[36]. In this study, we sought to determine the effect of DNA-PKcs on regulating c-Myc protein stability and focused on the involvement of Akt and GSK3 in its mechanistic pathway using the cell model with siRNA-silenced DNA-PKcs, DNA-PKcs deficient cells. Moreover, we have confirmed the effect of DNA-PKcs on regulating c-Myc stability in an overexpressed DNA-PKcs cell model, generated by sub-chronically exposing normal human liver L02 cells to very low dose of carcinogen 2,3,7,8-tetrachlorodibenzo-p-dioxin (TCDD).

## Materials and methods

### Cell culture and siRNA transfection

HeLa, HeLa-NC, HeLa-H1, HepG2, HepG2-NC, HepG2-H1, HepG2-H3, M059K (DNA-PKcs efficient human glioma cells), M059J (DNA-PKcs deficient human glioma cells), normal human liver L02 cells, ATM-deficient AT5BIVA, and human normal foreskin fibroblast HFC cells were maintained in Dulbecco modified Eagle medium (DMEM) containing 10% fatal bovine serum, 100 U/ml of penicillin and 100 μg/ml of streptomycin in a humidified chamber at 37°C in 5% CO_2_. M059K and M059J cell lines were kindly provided by Dr David Chen from the Department of Radiation Oncology, UT Southwestern Medical Center. HeLa-H1, and HeLa-NC were generated from HeLa cells, and HepG2-H1, HepG2-H3, HepG2-NC were generated from HepG2 cells, by stably transfecting with specific siRNA constructs targeting the DNA-PKcs catalytic motif (nucleotides 11637~11655, H1) or translation initiation region (nucleotides 354~372, H3) and a control construct (NC), respectively [35].

For experiments in which DNA-PKcs, GSK3α or GSK3β was knock-down transiently, the siRNA molecules were synthesized and purified by Cenechem company (Shanghai, China), including DNA-PKcs specific siRNA (5'-GGGCGCUAAUCGUACUGAAdtdt-3'), GSK3α specific siRNA (sense strand: 5'-CAUUCUCAU CCCUCCUCACdtdt-3'), GSK3β specific siRNA (sense strand: 5'-GAGCAAAUCAGAGAAAUGAdtdt-3'), and the non-specific control siRNA (sense strand: 5'-UUCUCCGAACGUGUCACGUdtdt-3'). For siRNA transfection, 1 × 10^5 ^cells were plated in each well of 6-well culture plate, 24 h later 30 μl of Lipofectamine 2000 reagent (Invitrogen, Carisbad, CA) was added into 1.5 ml DMEM without antibiotics and serum and incubated at room temperature for 5 min (solution A). A certain amount of siRNA was added into 1.5 ml DMEM without antibiotics and serum (solution B). Solution A and solution B were mixed and incubated at room temperature for 20 min. The medium in the cell culture was removed, and then 0.5 ml of Lipofectamine 2000-siRNA mixture and 1.5 ml of fresh DMEM without antibiotics were added to each culture well and gently mixed. After 48 hours of incubation, the cells were harvested for immunoblotting analysis.

### Antibodies

All antibodies were purchased commercially: anti-DNA-PKcs (H-163, Santa Cruz, CA), anti-Ku70 (H-308, Santa Cruz, CA), anti-c-Myc (9E10, Santa Cruz, CA), anti-phospho-c-Myc (Thr58/Ser62, #9401, Cell signal, Danvers, MA), anti-β-actin (I-19-R, Santa Cruz, CA), anti-Ubiquitin (P4D1, Cell signal, Danvers, MA), anti-Akt (#9272, Cell signal, Danvers, MA), anti-phospho-Akt (Ser473, #9271, Cell signal, Danvers, MA), anti-GSK3α (#9338, Cell signal, Danvers, MA), anti-GSK3β (#9332, Cell signal, Danvers, MA), anti-phospho-GSK3β(Ser9, #9336, Cell signal, Danvers, MA), anti-Rabbit IgG(H+L)/HRP (ZB-2301, Zhongshan, Beijing, China), and anti-Mouse IgG(H+L)/HRP (ZB-2305, Zhongshan, Beijing, China).

### 2,3,7,8-tetrachlorodibenzo-p-dioxin (TCDD) treatment

Normal human liver L02 cells were exposed to 0.01, 0.1, or 1.0 pM TCDD in the growth medium for 48 hours or 2 or 4 weeks. During the period of TCDD sub-chronic treatment, cells were subcultured for each 3–4 days. After ending of TCDD exposure, cells were subjected to growth curve and immunoblotting analyses under normal culture conditions.

### Cell growth and radiosensitive analyses

5×10^3 ^cells per well were seeded in 24-well culture plates The cell numbers from three wells were counted every day after plating for each group. Three independent experiments were performed, and the means were used to depict the growth curve.

In the radiosensitive experiment, cells were trypsinized, counted, and diluted to certain concentrations. Cell suspensions were irradiated immediately at room temperature using a ^60^Co γ-ray source at a dose rate of 2 Gy/min. Corresponding controls were sham irradiated. A colony-forming assay was performed immediately after irradiation by plating an appropriate number of cells (3 × 10^2 ^to 1 × 10^4^) into 60 mm diameter Petri dishes, in triplicate. After two weeks in culture, cells were fixed with methanol, stained with Giemsa solution, and colonies consisting of more than 50 cells were counted. After correction with plating cell numbers, the data of survival colonies were used to plot survival curves.

### Immunoblotting analysis and coimmunoprecipitation (CoIP)

The cells were harvested and washed twice in ice-cold phosphate buffered saline. Cell pellets were treated with lysis buffer (50 mmol/L Tris-HCL, pH 7.5, 1% Noridet P40, 0.5% Sodium deoxycholate, 150 mmol/L NaCl, 1 piece of protease inhibitor cocktail tablet in 50 ml solution), and the total protein was isolated. Protein (50 μg) was resolved on SDS/PAGE (8%), and then transferred onto the polyvinylidene fluoride (PVDF) membrane for immunoblotting detection.

In the CoIP experiment, HeLa-H1 and HeLa-NC cells were treated with 20 μM proteasome inhibitor MG132 (Z-Leu-Leu-Leu-al) (Sigma, Saint Louis, MO) for 2 h, or 40 mM GSK3 β inhibitor LiCl (ACROS, NJ) or 40 mM KCl (control) for 45 min before cell lysis. Coimmunoprecipitation was performed by using the Immunoprecipitation Kit (Protein A/G, Roche Molecular Biochemicals) according to the manufacturer's instructions. Briefly, cells were washed twice with ice-cold PBS and collected by centrifugation. The cell pellets were resuspended in pre-chilled lysis buffer (50 mM Tris-HCl, pH 7.5, 150 mM NaCl, 1% Nonidet P40, 0.5% sodium deoxychoolate and certain amount of complete tablet provided by the Kit) and homogenized. The supernatants were collected by centrifugation at 12 000 × *g *for 10 min at 4°C to remove debris, and then subjected to immunoprecipitation. After precleared with protein A/G-agarose, the supernatants were reacted for 3 h with 2 μg of anti-c-Myc antibody at 4°C followed by overnight incubation with protein A/G-agarose at 4°C. The immunoprecipitates were collected by centrifugation, and washed twice with washing buffer 1 (50 mM Tris-HCl, pH 7.5, 500 mM NaCl, 1% Nonidet P40 and 0.05% sodium deoxychoolate), and one time with washing buffer 2 (10 mM Tris-HCl, pH 7.5, 0.1% Nonidet P40 and 0.05% sodium deoxychoolate). The immunoprecipitates were denatured by heating to 100°C for 3 min in gel-loading buffer and centrifuged at 12 000 × *g *for 20 s to remove the protein A/G-agarose. The denatured proteins were resolved by 8% SDS-PAGE and subjected to immunoblotting analysis with the anti-ubiquitin antibody.

### Determination of c-Myc protein stability

HeLa-H1 and HeLa-NC cells were pretreated with 20 μM MG132 (Sigma, Saint Louis, MO) for 2 h to accumulate protein, then washed with cold-PBS three times to remove the MG132, followed by treatment with 40 μg/ml cycloheximide (CHX) (Sigma, Saint Louis, MO) at 37°C to block novel protein synthesis. The cells were harvested at the given times after CHX treatment, and subjected to immunoblotting analysis with the anti-c-Myc antibody.

## Results

### DNA-PKcs modulates c-Myc protein expression and cell proliferation

**Figure 1:**
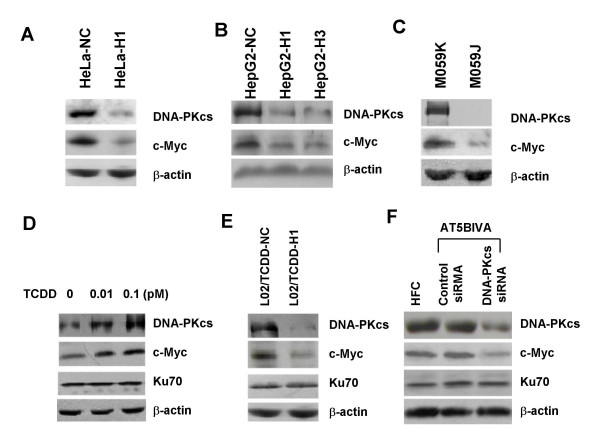
**DNA-PKcs regulates c-Myc protein expression (Immunoblotting analysis)**. (A) DNA-PKcs and c-Myc expression in the passage 35 of HeLa-H1 cells transfected with DNA-PKcs specific siRNA construct or HeLa-NC transfected with nonspecific siRNA construct (the same in all figures for HeLa-H1 and HeLa-NC cells). (B) DNA-PKcs and c-Myc expression in HepG2 cells transfected with DNA-PKcs specific siRNA construct (HepG2-H1 and HepG2-H3) or nonspecific siRNA construct (HepG2-NC). (C) Comparison of DNA-PKcs and c-Myc expression between human glioma M059K cells (DNA-PKcs efficient) and M059J cells (DNA-PKcs deficient). (D) Alterations of DNA-PKcs and c-Myc expression in normal human liver L02 cells after exposed to 0, 0.01 and 0.1 pM of TCDD for 4 weeks. (E) Effect of re-depressing TCDD-upregulated DNA-PKcs on c-Myc expression in L02 cells. L02/TCDD-H1 and L02/TCDD-NC cells were generated by exposing L02 cells to 0.1 pM TCDD for two weeks, then transfected with DNA-PKcs specific siRNA or nonspecific siRNA constructs, respectively. Cells grew in normal growth medium without TCDD after primary two weeks of TCDD exposure. (F) c-Myc protein expression in ATM-deficient AT5BIVA cells and human HFC fibroblasts, and the effect by siRNA-mediated suppression of DNA-PKcs.

We have firstly generated a number of cell models, including HeLa-H1, HepG2-H1, HepG2-H3, by silencing DNA-PKcs in human cervix cancer HeLa cells or liver cancer HepG2 cells respectively, with specific siRNA molecules targeting different position of DNA-PKcs sequence. The results indicated that siRNA-mediated silencing of DNA-PKcs led to stable downregulation of c-Myc protein, and even after 35 passages of subculture, the DNA-PKcs silenced HeLa-H1 cells expresses a lower level of c-Myc protein (Fig. 1A). Here we further shows that silencing of DNA-PKcs in HepG2 cells by siRNA strategy also leads to a significant depression of c-Myc oncoprotein (Fig. 1B). M059K and M059J cells are a pair of cell lines derived from the same malignant glioma specimen, M059K cells express functional DNA-PKcs, whereas M059J cells lack DNA-PKcs expression. This pair of cell lines offers a useful model for studying the biological function of DNA-PKcs. Therefore, we compared the c-Myc protein levels between these two cell lines and found that c-Myc level in M059J cells which lack DNA-PKcs was significantly lower than in M059K cells (Fig. 1C).

2,3,7,8-tetrachlorodibenzo-p-dioxin (TCDD) is a chemical carcinogen. We found that sub-chronic exposure of normal human liver cells L02 with 0.01 – 1.0 pM low dose of TCDD for 2 or 4 weeks dramatically increased DNA-PKcs expression. Interestingly, c-Myc protein level was concurrently augmented (Fig. 1D). We then asked whether depressing DNA-PKcs by siRNA strategy could alter c-Myc protein expression in this TCDD-treated L02 cells model. As shown in Fig. 1E, re-depressing DNA-PKcs by transfecting DNA-PKcs specific siRNA vector into L02/TCDD-H1 cells downregulated c-Myc protein, as compared with the control L02/TCDD-NC cells which were transected with the control non-specific siRNA vector. It has been demonstrated previously that DNA-PKcs deficiency causes down-regulation of ATM, another member of the phosphatidylinositol 3-kinase-related kinase (PIKK) family [37]. In order to investigate whether the involvement of DNA-PK in regulating c-Myc expression was due to further inhibition of ATM, we compared the c-Myc protein levels in ATM-deficient AT5BIVA cells and in normal HFC fibroblasts. As shown in Fig. 1F, there is no difference on c-Myc protein level between AT5BIVA cells and HFC cells. The involvement of DNA-PKcs in regulating c-Myc protein was further addressed with siRNA-mediated suppression of DNA-PKcs in AT5BIVA cells. Transient transfection of AT5BIVA cells with siRNA targeting DNA-PKcs resulted in a substantial decrease in DNA-PKcs protein level (Fig. 1F). Moreover, this suppression of DNA-PKcs was accompanied by a markedly decrease of c-Myc protein level in ATM5IBV cells. Taken together, our data illustrated the specific role of DNA-PKcs in regulating cellular c-Myc protein level.

**Figure 2:**
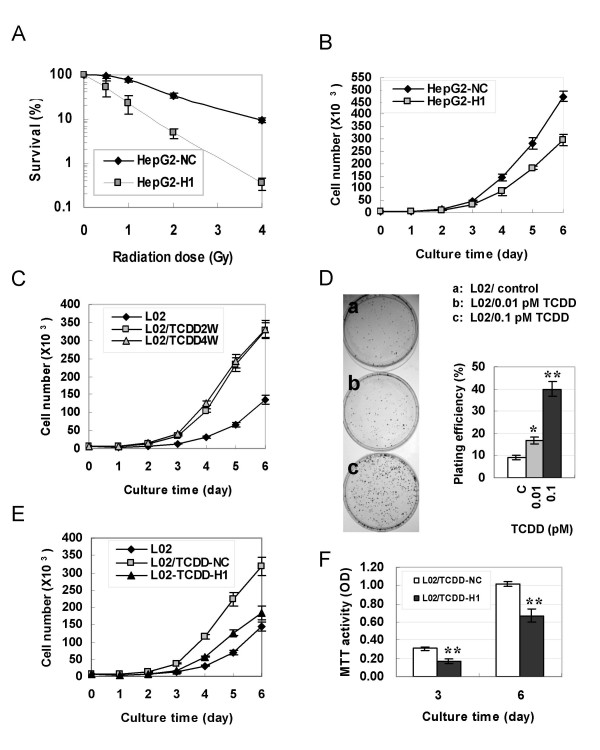
**Effects of DNA-PKcs expression status on cell proliferation and radiosensitivity**. (A) Survival curves of HepG2-H1 and HepG2-NC cells after γ-ray irradiation. (B) Growth curves of HepG2-H1 and HepG2-NC cells. (C) Growth curves of L02 cells measured after exposed to 0.1 pM TCDD for 2 weeks (L02-TCDD2W) or 4 weeks (L02-TCDD4W). (D) Focus colony formation assay. After treatment with 0, 0.01, or 0.1 pM of TCDD for two weeks, 1000 L02 cells were seeded into 60 mm diameter Petri dishes and cultured in normal growth medium without TCDD for 10 days, then fixed with methanol and stained with Giemsa solution. (E) Growth curves of L02/TCDD-H1 and L02/TCDD-NC cells, which were generated from L02 cells exposed to 0.1 pM TCDD for two weeks, then transfected with DNA-PKcs specific siRNA or nonspecific siRNA constructs, respectively. Cells grew in normal growth medium without TCDD after primary two weeks of TCDD exposure. (F) Cell proliferation activity as measured by MTT method. Student *T *– test: * *P *< 0.05; ** *P *< 0.01.

We then investigated whether the alteration of DNA-PKcs expression affects cell proliferation. The clonogenic survival assay showed that siRNA-mediated depression of DNA-PKcs strikingly sensitized HepG2-H1 cells to ionizing radiation (Fig. 2A). The killing effect of 2Gy irradiation was ~12 times higher on HepG2-H1 cells than on control HepG2-NC cells. Importantly, the growth curve analysis demonstrated that silencing DNA-PKcs decreased the proliferation activity of HepG2 cells (Fig. 2B) as well as HeLa cells (data not shown). It is well documented that exposure to some chemical toxins at very low doses can induce a stimulating effect (hormesis) on cell growth [38,39]. We demonstrated here that sub-chronic exposure to 0.01 – 0.1 pM of TCDD for 2 or 4 weeks drove human liver cells L02 to proliferate a great deal faster (Fig. 2C). The colony formation assay also showed that the colony focus size of low dose TCDD-treated cells was larger than that of control cells under the same culture conditions (Fig. 2D). The plating efficiency of TCDD-treated cells was also significantly increased as compared with control cells (Fig. 2D), while more than 1 pM concentration of TCDD exhibits a depression effect on cell proliferation (data not shown). Moreover, re-depression of DNA-PKcs in TCDD-treated L02 cells by specific-siRNA molecules not only downregulates c-Myc protein (Fig. 1E), but also re-modulates the proliferation rate to control levels (Fig. 2E,F).

### DNA-PKcs affects ubiquitination and stability of c-Myc protein

**Figure 3:**
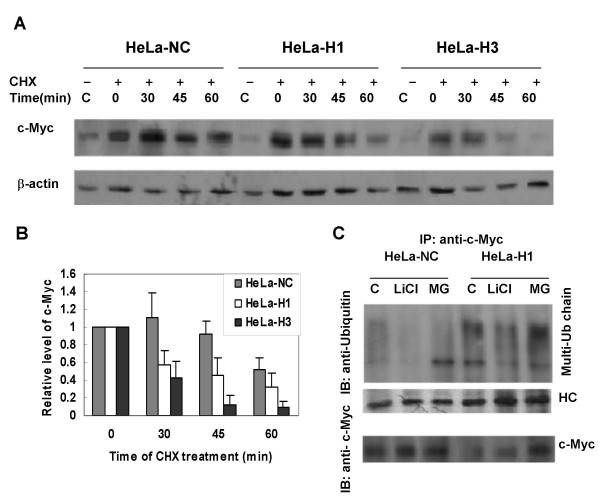
**DNA-PKcs regulates c-Myc stability**. (A) Silencing DNA-PKcs promoted c-Myc destruction. Cells were treated with cycloheximide for the given times to inhibit novel protein synthesis, then the cell extracts were prepared, followed by immunoblotting analysis of c-Myc. (B) Quantification of c-Myc destruction following CHX treatment based on densitometrical scanning of the immunohybridization signals of c-Myc protein shown in panel A. The data are the means with standard deviation from three independent experiments. (C) Silencing DNA-PKcs promoted ubiquitination of c-Myc. CoIP product (IP) of c-Myc was detected by immunoblotting (IB) with anti-ubquitin antibody (upper panel), while 1/2 the amount of c-Myc immunoprecipitates were subjected to immunoblotting analysis with c-Myc antibody as a control (lower panel).

To investigate the stability of c-Myc protein, the half-life time of c-Myc was analyzed by measuring c-Myc protein level alteration after blocking protein synthesis with cycloheximide (CHX). We have compared the c-Myc protein levels between DNA-PKcs-depressed cells and the control cells at 0, 30, 45 and 60 min after CHX treatment. In control HeLa-NC cells, c-Myc protein level decreased only after CHX treatment for more than 60 min. In contrast, c-Myc protein level decreased about 50% at 30 min after CHX treatment in DNA-PKcs silenced cells (HeLa-H1 and HeLa-H3) (Fig. 3A &3B). We next detected c-Myc ubiquitination, and found that silencing of DNA-PKcs in HeLa-H1 cells promotes the ubiquitination of c-Myc protein (Fig. 3C). Treatment with LiCl, an inhibitor of GSK3 kinase, impedes c-Myc ubiquitination. This result makes it likely that the downregulation of c-Myc protein by silencing DNA-PKcs is associated with the augmentation of c-Myc ubiquitination.

### GSK3 activity is necessary for the regulation of DNA-PKcs on c-Myc protein stability

**Figure 4:**
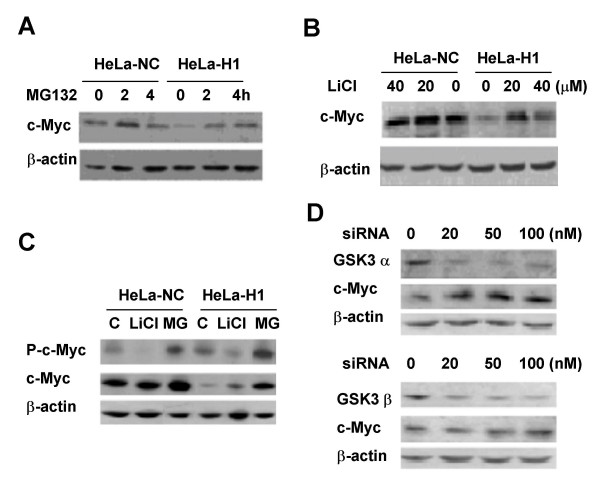
**GSK3 plays a role in the regulation of DNA-PKcs on c-Myc**. (A) Proteasome inhibitor MG132 recovered the downregulation of c-Myc in HeLa-H1 cells. (B) GSK3 isoforms inhibitor LiCl rescued the downregulated c-Myc in HeLa-H1 cells. (C) Phophorylation level of c-Myc (Thr58/Ser62) in HeLa-NC and HeLa-H1 cells, and the inhibiting effect of LiCl. (D) Depression of GSK3 α (upper panel) or GSK3 β (lower panel) by siRNA promotes c-Myc accumulation in HeLa-H1 cells.

The proteasome inhibitor MG132 remarkably increases c-Myc protein level in DNA-PKcs silenced HeLa-H1 cells (Fig. 4A). Moreover, the GSK kinase inhibitor LiCl can also recover c-Myc protein levels in HeLa-H1 cells (Fig. 4B). Phosphorylation of c-Myc on T58, which is catalyzed by GSK3 β kinase, is necessary for activating the ubiquitin/proteasome pathway to destroy c-Myc protein. Although total c-Myc levels in HeLa-H1 cells is lower than that in HeLa-NC cells, the base level of phosphorylated c-Myc protein in HeLa-H1 cells is higher than that in HeLa-NC cells (Fig. 4C). The accumulated c-Myc protein in MG132-treated HeLa-H1 cells exhibits a higher phosphorylation level as compared to HeLa-NC cells. Inhibition of GSK3 activity by LiCl dramatically decreases the level of phosphorylated c-Myc protein, but increases the accumulation of c-Myc protein in HeLa-H1 cells (Fig. 4C). In addition, the level of c-Myc protein is increased by siRNA-mediated depression of GSK3 α or β (Fig. 4D). These results demonstrate that CSK3 plays an important role in c-Myc stabilization modulated by DNA-PKcs.

### Phosphorylation level of Akt and GSK3 proteins is associated with DNA-PKcs expression status

**Figure 5:**
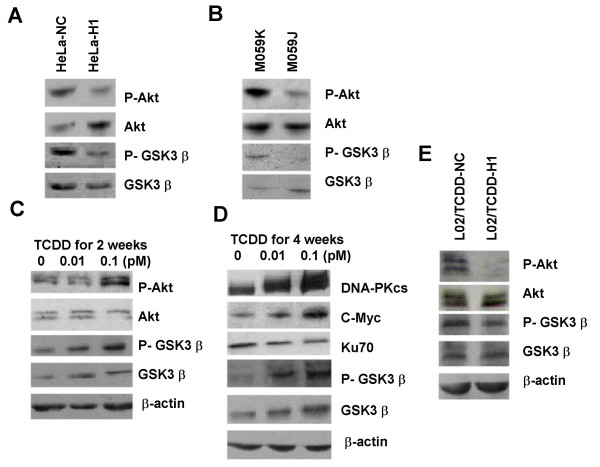
**DNA-PKcs controls phophsporylation of Akt and GSK3 β**. (A) Phosphorylation of Akt and GSK3 β was depressed in HeLa cells transfected with a DNA-PKcs specific siRNA construct (HeLa-H1). (B) Phosphorylation level of Akt and GSK3 β was lower in DNA-PKcs deficient M059J cells than in DNA-PKcs efficient M0591K cells. (C) & (D) Subchronic exposure to low dose TCDD for 2 weeks (C) or 4 weeks (D) upregulated the phosphorylation level of Akt and GSK3 β along with increased expression DNA-PKcs and c-Myc. (E) Depression of TCDD-increased DNA-PKcs by DNA-PKcs specific siRNA led to decreased phosphorylation level of Akt and GSK3 β. L02/TCDD-H1 and L02/TCDD-NC cells were described in Fig. 2(D).

We detected the phosphorylation level of Akt using the antibody against phosphorylated Akt on Ser-473 in the cell lines with different status of DNA-PKcs expression. It is clear that the level of phosphorylated Akt in DNA-PKcs silenced HeLa-H1 cells as well as DNA-PKc deficient M059J cells is dramatically lower than that in the control cells (Fig. 5A &5B). Consequently, the phosphorylation level of GSK3 β, a substrate of activated Akt, is simultaneously decreased in the cells lacking DNA-PKcs. Conversely, the phosphorylation of Akt and GSK3 β in normal liver L02 cells is augmented, along with the increasing of DNA-PKcs and c-Myc proteins, as the consequence of sub-chronic exposure with a low dose of TCDD (Fig. 5C &5D). We then investigated that whether re-depression of TCDD-augmented DNA-PKcs expression in L02 cells affects the phosphorylation of Akt. We found that transfection with DNA-PKcs specific siRNA not only downregulated the TCDD-augmented DNA-PKcs and c-Myc expression in L02 cells, but also led to a decrease in the phosphorylation of Akt and GSK3 β (Fig. 5E). Importantly, the increased proliferation activity by a low dose of TCDD exposure was also reversed to the level of control cells (Fig. 2E). These results indicated that Akt/GSK3 signaling is involved in the pathway through which DNA-PKcs modulates c-Myc stability.

## Discussion

C-Myc is intimately involved in cell proliferation [1,2], carcinogenesis [3], tumor progression [40,41], angiogenesis [42] and metastasis [43]. Dysregulated accumulation of c-Myc protein is often observed in a variety of human cancers [4-9]. This abnormal accumulation of c-Myc in human cancers can be attributed to multiple causes, for instance, gene translocation and amplification [2,4,40,41,43,44], gene mutations on hot spots, *e.g.*, Thr58 which abolishes c-Myc phosphorylation and results in decreased ubiquitination and proteasome-mediated degradation of c-Myc [7,18,19], and dysregulation of the mechanistic signaling pathway controlling c-Myc stability. In the present study, we highlighted the overexpression of DNA-PKcs and its role in controlling the stability of the oncoprotein c-Myc. Our study demonstrates that DNA-PKcs expression status in cells is closely associated with c-Myc protein levels.

Recently, overexpression of DNA-PKcs was reported in various human tumors [24-30,45]. For example, Hosoi et al. have detected the expression of DNA-PK in tumor tissues and adjacent normal tissues of 12 colorectal cancers, and found that the activity and expression level of DNA-PKcs were significantly higher in tumor tissues than in normal tissues [24]. Um et al. have revealed increased protein level and activity of DNA-PKcs in the metastatic cancer cell lines as compared with their parental cells, and suggested that the activities of DNA-PK as well as EGFR are associated with the metastatic phenotype [45]. We have assessed DNA-PKcs in 47 cases of liver neoplasm by immunohistochemistry, and found a wide variation in the expression levels of DNA-PKcs among different types of liver neoplastic tissues. The highest expression was detected in hepatocellular carcinoma, followed by cholangioadeno carcinoma and biliary cystadeno-carcinoma. Relatively weak expression was detected in papillary adenoma cases, but clearly increased expression was observed in cases of papillary adenoma with hyperplasia or infiltration. However, very weak immunohistochemical staining was detected in the adjacent normal tissues [29]. It is believed that a suitable base level of DNA-PKcs in cells is necessary for maintaining the genomic stability via its role in DNA repair. However, the biological significance of overexpressed DNA-PKcs in cancer cells, besides its potential effect of increasing resistance of cancer cells to radiotherapy or chemotherapy, has attracted our attention. Our results indicate that silencing DNA-PKcs of HeLa cells causes not only an increased sensitivity to ionizing radiation (Fig. 2A), but also a decrease in proliferation (Fig. 2B). More interestingly, the increased proliferation of normal human liver L02 cells induced by sub-chronically exposing to low dose of carcinogen TCDD is associated with the overexpression of DNA-PKcs induced by TCDD (Fig. 2C,D). These results suggest that overexpressed DNA-PKcs plays a role in promoting cell proliferation and even has transforming potential. We have previously reported that silencing DNA-PKcs alters the expression of a set of genes functionally related to proliferation and differentiation, some of which are c-Myc target genes, e.g. p21, p27, NDRG1 [36]. Moreover, siRNA-medicated silencing of DNA-PKcs results in downregulation of c-Myc protein in HeLa cells [35]. Here we further demonstrated that c-Myc protein levels in malignant glioma M059J cells lacking DNA-PKcs is much lower than that in M059K cells expressing DNA-PKcs. In addition, overexpressing DNA-PKcs in normal liver L02 cells by sub-chronic exposure to a low dose of TCDD simultaneously leads to an increased c-Myc level. This increased c-Myc level was re-downregulated along with the depression of DNA-PKcs mediated by siRNA strategy. Therefore, we offered evidence suggesting a novel biological role for DNA-PKcs, which is potentially associated with cell proliferation and transformation through controlling c-Myc protein levels, beyond its well-defined function as a component involved in DNA double-strand break repair and V(D)J recombination.

Silencing of DNA-PKcs does not alter the level of c-Myc mRNA in the cells [35]. Therefore, we suggest that DNA-PKcs regulates cellular c-Myc protein levels possibly by affecting the stabilization of c-Myc protein. Phosphorylation of c-Myc protein on Thr58 and Ser62 is essential for the ubiquitin-proteasome pathway of c-Myc destruction. Phosphorylation on Thr58 by GSK3 regulates the binding of Fbw7 to c-Myc, triggering c-Myc ubiquitination and destruction [15]. Our data show that silencing DNA-PKcs leads to increased ubiquitination and decreased half-life of c-Myc protein (Fig. 3). The phosphorylation of c-Myc on Thr58 was also increased (Fig. 4C). These data suggest that DNA-PKcs does regulate the stabilization of c-Myc protein by affecting its phosphorylation on Thr58 and ubiquitination.

DNA-PKcs was previously reported to phosphorylate Akt on Ser473 [46], while Akt phosphorylates and inactivates GSK3 β [47]. It is likely that DNA-PKcs regulates c-Myc stability via phosphorylation of Akt, which in turn inactivates GSK3 β, resulting in stabilization of c-Myc. Therefore, we have further analyzed the possible link between DNA-PKcs and GSK3 β/c-Myc by investigating the role of Akt. Here we observed that silencing DNA-PKcs or DNA-PKcs deficiency caused deceased phosphorylation of Akt on Ser473 as well as GSK3 β (Fig. 5A&5B). Inhibition of GSK3 β by its inhibitor LiCl or specific siRNA rescued the downregulated c-Myc protein mediated by silencing DNA-PKcs (Fig. 4B &4D). As indicated above, we have established a cell model with an increased DNA-PKcs expression by sub-chronically exposing normal liver L02 cells with 0.1 pM low dose of carcinogen TCDD (Fig. 1C). c-Myc protein level as well as phosphorylation of Akt and GSK3 β is also increased in this cell model (Fig. 5C &5D). Most importantly, re-silencing DNA-PKcs by siRNA strategy resulted in downregulated c-Myc (Fig. 1D) as well as decreased phosphorylation of Akt and GSK3 β (Fig. 5E) in this cell model. Thus, it is conceivable that Akt and GSK3 β are involved in the mechanistic pathway by which DNA-PKcs regulates c-Myc stability.

## Conclusion

Given the facts that DNA-PKcs is a DNA damage sensing protein in responding to environmental genotoxic or oxidative stresses in cells and overexpression of DNA-PKcs frequently occurs in human cancers, long-term exposure of cells to genotoxins or oxidative stress, e.g. TCDD which produces oxidative stress in cells [48], may lead to constitutive overexpression of DNA-PKcs as the consequence of persistently stressing reactions. We suggest that overexpressed DNA-PKcs is another critical cause contributing to the stabilization of c-Myc oncoprotein via the Akt/GSK3 β pathway. Taken together, a suitable level of DNA-PKcs in cells is necessary for maintaining genomic stability via its DNA repair function, while dysregulated overexpression of DNA-PKcs may contribute to cell proliferation and even oncogenic transformation via stabilizing the c-Myc oncoprotein. The environmental genotoxic or oxidative stresses could be one of the causes destroying the balance of DNA-PKcs expression.

## Competing interests

The authors declare that they have no competing interests.

## Authors' contributions

JA, DYY and QZX performed experiments and analyzed data, and they contributed equally to this work. SMZ and YYH performed the experiment of siRNA-mediated depression of GSK3 β and its effect on c-Myc protein expression. ZFS and YW constructed vectors and performed gene transfection. DCW and PKZ designed the experiments, analyzed data, and wrote the manuscript. All authors read and approved the final version of the manuscript.
